# Prenatal dexamethasone exposure alters effort decision making and triggers nucleus accumbens and anterior cingulate cortex functional changes in male rats

**DOI:** 10.1038/s41398-022-02043-4

**Published:** 2022-08-19

**Authors:** Ana Verónica Domingues, Bárbara Coimbra, Raquel Correia, Catarina Deseyve, Natacha Vieitas-Gaspar, Stan B. Floresco, Nuno Sousa, Carina Soares-Cunha, Ana João Rodrigues

**Affiliations:** 1 Life and Health Sciences Research Institute (ICVS), School of Medicine, University of Minho, Braga, Portugal; 2 ICVS/3B’s-PT Government Associate Laboratory, Braga/Guimarães, Portugal; 3 Department of Psychology and Djavad Mowafaghian Centre for Brain Health, University of British Columbia, 2136 West Mall, Vancouver, BC V6T 1Z4 Canada; 4 Clinical Academic Center-Braga (2CA), Braga, Portugal

**Keywords:** Molecular neuroscience, Learning and memory

## Abstract

Daily, individuals select actions based on cost-benefit to allocate resources into goal-directed actions. Different brain regions coordinate this complex decision, including the nucleus accumbens (NAc), anterior cingulate cortex (ACC), and ventral tegmental area (VTA). *In utero* exposure to synthetic glucocorticoids (iuGC), such as dexamethasone, triggers prominent motivation deficits but the impact of this exposure in the ACC-NAc and/or ACC-VTA circuits is unknown. Here, we show that iuGC exposure causes decreased motivation for natural rewards (food) and impaired effort-based decision-making. Importantly, reduced neuronal activation (number of c-fos^+^ neurons) was observed in the NAc core and ACC of iuGC rats in comparison to CTR rats after performing the effort-based decision-making task. In addition, iuGC treatment led to increased NAc and ACC basal neuronal activity. Electrophysiological recordings during optogenetic modulation of ACC terminals in the NAc revealed that the ACC-NAc circuit is dysfunctional in iuGC animals. These data suggest that iuGC animals present motivational and effort-based decision-making deficits that can be associated with the observed ACC-NAc dysfunction.

## Introduction

Exposure of individuals to stressful events during development has been for long considered as a contributing factor for the development of psychiatric disorders such as depression, schizophrenia, or drug addiction later in life [1–5]. These *programming effects* are proposed to occur through abnormal release of stress hormones, glucocorticoids (GC), that are potent modulators of neuronal structure and function [4, 6]. Synthetic GC, such as dexamethasone (DEX), are widely used in the clinics during risk of pre-term labor gestations to promote fetal lung maturation [7]. Importantly, DEX is able to cross the maternal-placental barrier to a greater extent than endogenous GC, which can lead to additional risks for the developing brain [8, 9]. Although the pathophysiology of these disorders is distinct, they share a key denominator: dysfunction of the mesocorticolimbic dopaminergic pathway [10–12].

The mesocorticolimbic system is central for reward-related behaviors and motivation. Dopaminergic projections arising from the ventral tegmental area (VTA) to the nucleus accumbens (NAc), as well as to prefrontal cortical brain areas, are key mediators of the hedonic impact of rewards, reinforcement learning, decision making processes, and effort-dependent behaviors [13–15]. In fact, NAc dopamine depletion/antagonism reallocates responses to lower cost alternatives, reducing motivation [16–18]. In addition, reciprocal neuronal projections arising from sub-nuclei of the prefrontal cortex (PFC), such as the anterior cingulate cortex (ACC), are also critical in these fundamental behavioral processes. The ACC has partially dissociable roles in several dimensions of decision making [19], which include evaluation of the expected costs and benefits associated with each potential choice of action during decision making. Importantly, increased ACC-VTA connectivity has been implicated in anticipatory value-based decision making, while increased VTA-ACC signaling is predictive of subsequent choice adaptation [20]. Also, ACC-NAc projections have been reported to play a key role in mediating reward processing, since subsequent lesion of ACC and NAc, using an asymmetric procedure, results in impaired Pavlovian approach behavior [21]. These results highlight that the ACC, NAc, and VTA are nodes of a corticostriatal circuit involved in reward processing that, if disrupted, may underlie motivational deficits.

We have previously shown that *in utero* exposure to increased levels of the synthetic GC DEX (iuGC-exposed rats) targets the mesolimbic dopaminergic system, causing changes in the NAc [22, 23] and mPFC [24, 25]. This phenotype is accompanied by decreased levels of dopamine and dopamine receptors in the NAc and mPFC, as well as neuronal structural changes [22, 23]. We observed a significant decrease in volume and total cell numbers in both NAc core and shell sub-regions and changes in the number of spines in medium spiny neurons of the NAc shell [23]. Additionally, a significant decrease in the volume and total number of cells is also observed in the ACC of iuGC rats, which is accompanied by a significant decrease in the dendritic length of basal dendrites of pyramidal neurons [22]. Importantly, the overall hypodopaminergic state present in the brain of these animals results in increased proneness for reduced social behaviors [26], decreased motivational levels towards natural rewards [22, 27], and increased drug-seeking behavior [23].

However, it is still unclear if and how iuGC exposure alters the ACC-NAc and/or ACC-VTA circuit activity. Thus, herein, we assessed the effects of iuGC exposure on the performance of a classical motivation task and an effort-based decision-making task. We evaluated neuronal activation after task performance using c-fos counting, and performed electrophysiological recordings in vivo of the ACC, NAc, and VTA. We show that iuGC exposure decreases motivation towards natural rewards and perturbs effort-based decision-making, the latter being associated with a significant decrease in c-fos^+^ neurons in the NAc and ACC. Moreover, this behavioral impairment was accompanied by alterations in ACC-NAc neuronal activity in iuGC animals.

## Material and methods

### Animals and treatments

To generate the iuGC model, pregnant female Wistar Han rats (9–11 weeks old) were individually housed under standard laboratory conditions: artificial 12 h light/dark cycle (lights on from 08:00 to 20:00 h), with an ambient temperature of 21 ± 1 °C and relative humidity of 50–60%; breeding diet (4RF25, Mucedola SRL, Italy) and water were available *ad libitum*. Pregnant rats were subcutaneously injected with the synthetic GC DEX (Burlington MA, USA) at 1 mg kg^−1^ (iuGC animals) or vehicle (sesame oil, Sigma, Burlington MA, USA; CTR- control animals) on days 18 and 19 of gestation. Male offspring from 4 different litters were used for behavioral assessment (details of the model can be found in [10, 22, 23, 26, 28]). On postnatal day 21, progeny was weaned according to prenatal treatment and sex, and was housed in pairs. Male rats were maintained under standard laboratory conditions as described above, with the exception of the diet (standard diet 4RF21, Mucedola, Italy). For behavior assessment, animals with 3 months of age were used. Sample size used in behavioral tests was chosen according to previous studies using the same animal model [22, 23, 27]; no randomization method was used to determine how animals were allocated to experimental groups. The investigator was not blind to the group allocation during behavioral performance.

All manipulations were conducted in accordance with European Regulations (European Union Directive 2010/63/EU). Animal facilities and the people directly involved in animal experiments were certified by the Portuguese regulatory entity – Direção-Geral da Alimentação e Veterinária (DGAV #023432). All the experiments were approved by DGAV.

### Behavior

Two days before the beginning of behavioral experiments, rats were placed under food restriction to maintain 90% of their initial free-feeding weight (~7 g/day/animal of standard chow diet were given to each animal at the end of each training day). All behavioral sessions were performed during the light period of the light/dark cycle.

Rats were habituated to highly palatable high-sugar food pellets (dustless precision 45 mg food pellets; F0021; Bio-Serv, Flemington, NJ, USA) 1 day before training initiation, in their home cage, which were used as the reward during the behavioral protocol. Behavioral sessions were performed in operant chambers (Med Associates, St. Albans, USA) that contained a central magazine to provide access to food pellets, two retractable levers with cue lights above them that were located on each side of the magazine. Chamber illumination was obtained through a 2.8 W, 100 mA light positioned at the top-center of the wall opposite to the magazine. The chambers were controlled by a computer equipped with the Med-PC software (Med Associates, St. Albans, USA).

### Progressive ratio (PR) schedule of reinforcement

#### Continuous reinforcement (CRF) training

All training sessions started with illumination of the house light that remained ON until the end of the session. On the first CRF training sessions, one lever was extended throughout the session, and a single lever press would result in the delivery of a food pellet, with a maximum of 50 pellets earned within 30 min. In some cases, on the first two sessions, food pellets and food pellet dust were placed on the lever to promote lever pressing. After completion of the first CRF training session, rats (*n*_CTR_ = 10, *n*_iuGC_ = 12) were trained to lever press on the opposite lever using the same training procedure. In the five following days, the side of the active lever was alternated between sessions.

#### Fixed ratio (FR) training

Rats were then trained on a single session to lever press one time for a single food pellet in a FR 1 schedule consisting in 50 trials in which both levers are presented, but the active lever is signaled by the illumination of the cue light above it. FR sessions began with extension of both levers (active and inactive) and illumination of the house light and the cue light above the active lever. Completion of one lever press resulted in the delivery of one pellet, retraction of the levers and the cue light turning OFF for a 20 s intertrial interval (ITI). After FR1 training, rats were trained first with one lever active and then with the opposite lever active in separate sessions (in the same day). Rats were then trained using a FR4 reinforcement schedule (pressing 4 times in the active lever resulted in the delivery of 1 food pellet) for 4 days, a FR8 reinforcement schedule (pressing 8 times in the active lever resulted in the delivery of 1 food pellet) for 1 day followed by a FR4 reinforcement schedule for 1 day. This variable schedule would allow animals to expect flexibility in the number of lever presses necessary to receive a single food pellet.

#### PR test

On the following day, rats performed a PR session, that was similar to the FR sessions except that the number of lever presses required to obtain a single food pellet increased exponentially: on each trial (T) the required number of lever presses was the integer (rounded down) of 1.4^(T–1)^ lever presses, starting at 1 (i.e., 1, 1, 1, 2, 3, 5, 7, 10, 14, 20, 28, etc). The PR session ended after 1 h had elapsed or 15 min have passed without completion of the response requirement in a trial. The total number of lever presses, number of food pellets earned, and breakpoint (the last completed ratio) of the PR session were used for data analysis.

### Effort-discounting task

This protocol was performed in the same animals (*n*_CTR_ = 10, *n*_iuGC_ = 12) that performed the PR test, and was based on a protocol previously described [29, 30].

Rats were first trained on a simplified version of the full task, for 3 days. These sessions consisted of 90 trials and began with both levers retracted and the chamber in darkness. Every 40 s, a trial began with illumination of the house light and insertion of one of the two levers into the chamber. If the rat failed to respond on the lever within 10 s, the lever was retracted, the house light was turned OFF and the trial was scored as an omission. If the rat responded within 10 s, the lever retracted, a single pellet was delivered immediately, and the house light remained illuminated for another 4 s. In every pair of trials, the left or right lever was presented once, and the order within the pair of trials was random.

After initial training, animals received one daily 32 min session that consisted of 48 discrete choice trials, separated into four blocks. Each block of trials was comprised of two forced-choice trials on which only one lever was presented (one trial for each lever, in randomized order) followed by 10 free-choice trials. A session began in darkness with the levers retracted (the intertrial interval state – ITI). Trials began at 40 s intervals with the illumination of the house-light, followed by extension of one or both levers 3 s later. One lever was designated as the high-reward (HR) lever and the other the low-reward (LR) lever (counterbalanced left/right between animals), which remained constant for the duration of the experiment. If a rat did not respond within 25 s of lever presentation (omission), the chamber was reset to the ITI state. Responding on the LR lever caused both levers to be retracted and the immediate delivery of one pellet. Response on the HR lever caused the immediate retraction of the LR lever, while the HR remained inserted in the chamber to allow completion of the pressing requirement. To increase the effort requirement, rats had to complete a fixed ratio of presses on the HR lever to receive delivery of four reward pellets. Immediately after the last required lever press on the HR lever was achieved, the lever retracted, and four pellets were delivered. Pellets were delivered 0.5 s apart. After food delivery, the house-light remained on for another 4 s, after which the chamber returned to the ITI state.

The fixed ratio of lever presses required to obtain the HR was varied systematically across each block, starting at 2, then 5, 10, and 20 presses, respectively. If the rat chose the HR lever but did not complete the ratio within 25 s, the lever retracted, no food was delivered and the chamber reverted to the ITI state, although the animal’s choice was still incorporated into the data analysis. The test ended when the rat achieved a criterion performance of 75% of HR choice in FR2 during three consecutive days. CTR and iuGC groups reached the criteria in different days, so CTR rats ceased training before iuGC group.

For behavioral analysis, we divided the effort-discounting task in two parts: early and late learning. The first one represents the average of HR option on the day one and two of the task for each FR (2, 5, 10, and 20). Late learning corresponds to the average of HR option of the fifth and sixth days of the task. The days selected for the late learning were made taking in account that control group achieved criterion performance after six days.

### Immunohistochemistry (IHC)

CTR and iuGC animals that performed the effort-discounting task received 4 additional days of effort-discounting training. On the 4th day of effort-discounting, rats were deeply anesthetized with pentobarbital (Eutasil, Lisbon, Portugal), 60 min after the session to allow expression of c-fos protein, and transcardially perfused with saline followed by 4% paraformaldehyde. Brains were removed and sectioned coronally at a thickness of 50 μm on a vibrating microtome (VT1000S, Leica, Germany).

Free-floating sections were pre-treated with 3% hydrogen peroxide (H_2_O_2_) in phosphate buffered saline (PBS) for 30 min. After blocking using 5% fetal bovine serum (FBS) in PBS-Triton 0.3% for 1 h at room temperature, sections were incubated overnight at room temperature (20 °C) with the primary antibody anti-c-fos (1:750; ABE457; Millipore, MA, USA). Afterwards, sections were washed and incubated with the secondary polyclonal swine anti-rabbit biotinylated antibody (1:200, DAKO, Denmark) for 1 h, processed with an avidin-biotin complex solution (ABC-Elite Vectastain reagent; Vector Lab., USA) and detected with 0.5 mg ml^−1^ 3,3′-diaminobenzidine (Sigma, Burlington MA, USA) including 12.5 µl of 30% H_2_O_2_ as a substrate in Tris-HCL solution. Sections were washed and mounted on glass slides, air-dried, counterstained with Hematoxilin and coverslipped with Entellan (Merck, NJ, USA).

For each animal, 5 slices containing the regions of interest were used. The outline of the regions of interests (ROI), ACC (*n* = 5/group), NAc (*n*_CTR_ = 8; *n*_iuGC_ = 7) and VTA (*n*_CTR_= 8; *n*_iuGC_ = 9), was defined in each section using established landmarks according to Paxinos and Watson [31] atlas. C-fos^+^ cell numbers were obtained using a camera (PL-A622, PixeLINK, Ontario, Canada) attached to a motorized microscope (BX51TF, Olympus, Tokyo, Japan and Visiopharm Integrator System software (Visiopharm, Copenhagen, Denmark). Average cell numbers were estimated using the optical fractionator method as described elsewhere [32]. Briefly, a grid of virtual 3D-boxes (100 µm × 100 µm × 30 µm) was superimposed on the section of the ROI. An estimate of total number of cells was then derived from the number of cells falling inside the boxes (ACC and NAc 50% of the boxes were counted; VTA 100% boxes were counted), box spacing, and total number of boxes. Coefficients of error were automatically computed according to the formulas of Gundersen et al. [33] for cell numbers. For rostrocaudal axis analyses, we grouped sections into four rostrocaudal segments according to the Paxinos and Watson [31] atlas. For the ACC, the segments used were: 2.8–2.20, 2.0–1.70, 1.60–1.20 and 1–0.8; for NAc 2.5–2.2, 2.1–1.8, 1.7–1.3 and 1.2–1.0; and for VTA −4.8- −5.10, −5.20- −5.50, −5.7- −6.10 and −6.20- −6.50 (values refer to coordinates from bregma). The average of c-fos^+^ cell/mm^2^ for each segment was then plotted. The investigator performing cell countings was blinded to group.

### In vivo electrophysiological recordings and optogenetic stimulation

#### Surgeries

A second set of CTR and iuGC animals (*n* = 5/group) were anesthetized with 75 mg kg^−1^ ketamine (Imalgene, Merial, Lisbon, Portugal) plus 0.5 mg kg^−1^ medetomidine (Dorbene, Cymedica, Lisbon, Portugal). 0.5 μl of an AAV5-CAMKII-ChR2-YFP (UNC Vector Core, NC, USA; titer: 1 × 10^13^ GC ml^−1^, rate of injection 1 μl min^−1^) was unilaterally injected into the ACC -coordinates from bregma, according to Paxinos and Watson, 2006 [31]: +1.9 mm anteroposterior (AP), 0.5 mm mediolateral (ML), and 1.4 mm dorsoventral (DV). Four weeks after surgery, this set of rats were used for electrophysiological recordings.

#### Recordings

Rats were anaesthetized with urethane (1.44 g kg^−1^, Sigma, Burlington MA, USA); total dose was administered in three separate intraperitoneal injections, 15 min apart. Adequate anesthesia was confirmed by lack of withdrawal responses to hindlimb pinching. A recording electrode coupled with a fiber optic patch cable (Thorlabs, Newton, New Jersey, USA) was placed in the ACC (coordinates from bregma: +1.9 mm AP, 0.5 mm ML, and 1.4 mm DV), using a stereotaxic frame (David Kopf Instruments, Tujunga, CA, USA) with nontraumatic ear bars (Stoeling, Wood Dale, IL, USA). Other recording electrodes with fiber optic attached were placed in the NAc - coordinates from bregma: 1.2 mm AP, 1.2 mm ML, and 6.0–7.0 mm DV, and in the VTA - coordinates from bregma: 5.3 mm AP, 0.6 mm ML, and 7.5–8.3 mm DV.

Single neuron activity was recorded extracellularly with a tungsten electrode (tip impedance 5–10Mat 1 kHz) and data sampling was performed using a CED Micro1401 interface and Spike2 software (Cambridge Electronic Design, Milton, Cambridge, UK). A DPSS 473 nm laser system (CNI, Changchun, China), controlled by a stimulator (Master-8, AMPI, New Ulm, MN, USA) was used for intracranial light delivery. Optical stimulation was performed as follows: 473 nm; frequency of 20 Hz; 5 ms pulses over 1 s, 5 mW for ACC at the tip of the fiber, and 10 mW for NAc and VTA for ACC terminals stimulation. Average firing rate was calculated considering activity recorded during a 60 s interval, for each individual brain region. Firing rates recorded upon optogenetic stimulation were calculated for the baseline (60 s before stimulation), stimulation period (1 s), and after stimulation period (60 s after the end of stimulation), using a bin size of 1 s.

ACC neurons were classified according to previous descriptions [34–36]. Putative glutamatergic neurons were identified as having a broader action potential waveform (peak-to-valley) above 0.5 ms and a baseline firing rate lower than 10 Hz; putative interneurons were identified as having a waveform peak-valley lower than 0.3 ms and a baseline firing rate higher than 10 Hz.

NAc neurons were classified according to previous descriptions [37, 38]. In short, putative fast-spiking parvalbumin-containing interneurons (pFSs), were identified as having a waveform half-width of less than 100 ms and a baseline firing rate higher than 10 Hz; tonically active putative cholinergic interneurons (pCINs) were identified as those with a waveform half-width bigger than 300 ms. Putative MSNs (pMSNs) were identified as those with baseline firing rate lower than 5 Hz and that do not meet the wave form criteria for pCIN or pFS neurons.

Single units in the VTA were separated into putative dopaminergic (pDAergic) and putative GABAergic (pGABAergic). This classification was based on firing rate and waveform duration, previously validated by others, in which both electrophysiological properties and neurochemical composition (presence or absence of the tyrosine hydroxylase enzyme, only present in dopaminergic neurons) were described [39–41]. Cells presenting baseline firing rate lower than 10 Hz and a waveform duration higher than 1.1 ms were considered pDAergic neurons. Cells presenting baseline firing rate higher than 10 Hz and waveform duration lower than 1.1 ms were classified as pGABAergic.

### Statistical analysis

Statistical analysis was performed in GraphPad Prism 5.0 (La Jolla, CA, USA) and SPSS Statistics (Armonk, NY, USA).

Prior to any statistical comparison between groups, the presence of outliers through Tukey test was assessed. Outliers were excluded prior to statistical analysis. Figure legends report the final number of animals excluding outliers. Normality tests (Kolmogorov–Smirnov) were performed for all data analyzed, and appropriate statistical analysis was applied accordingly.

Parametric tests were used whenever Shapiro–Wilk normality test SW > 0.05. Non-parametric analysis (Mann–Whitney test) was used when normality of data was not assumed.

Two-way analysis of variance (ANOVA) was used to analyze learning session of the PR training sessions (CRF and FR) (factors used: Group (CTR vs iuGC) and day of training) and effort-based decision-making task (factors used: Group (CTR vs iuGC) and FR schedule (FR2, FR4, FR10, and FR20)). Bonferroni’s post hoc multiple comparison tests were used for group differences determination.

Statistical analysis between two groups was made using Student’s t-test (CTR *versus* iuGC), for the PR test session, for c-fos^+^ cell counting, and for average firing rate activity.

One way ANOVA for repeated measures was used to compare firing rate baseline, stim and after stim for all cell types of all brain regions assessed; Bonferroni’s post hoc multiple comparisons were used for group differences determination.

The degree of responsiveness during the effort-related task was measured by calculating the area under the curve for each group (AUC), using the trapezoid rule. To examine how our treatments altered the probability of achieving criterion performance in the task over time, we generated success curves, quantifying the percentage of HR preference during early learning (day 1 and 2) and late learning (day 5 and 6). Comparison between CTR and iuGC curves was made using the log-rank Mantel–Cox test. To calculate the number of days that each animal per group needed to fulfill the criteria of the effort discounting task, a survival curve was used. Animals’ survival curve was plotted using Kaplan–Meier survival curves and analyzed by log rank test. Pearson’s correlation was used to examine the relationship between recruited c-fos^+^ cells and effort-based task (% of HR choice in the FR2 schedule). Results are presented as mean ± SEM. Statistical significance was accepted for *p* < 0.05.

## Results

### iuGC exposure reduces motivation for food in a progressive ratio task

**Fig. 1:**
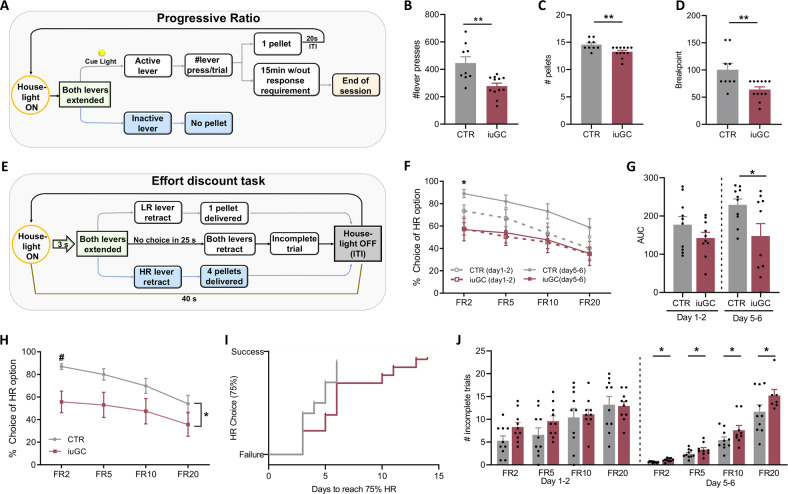
iuGC exposure impairs motivated behavior. **A** Progressive Ratio task scheme. **B** Total number of lever presses during PR test (*n*_CTR_ = 9, *n*_iuGC_ = 12). **C** Total number of pellets received in PR test session. **D** Animal’s breakpoint in the PR test. **E** Effort-discounting task scheme. **F** Percentage of HR preference during early learning (day 1 and 2) and late learning (day 5 and 6) during the effort-based task; * indicates difference between iuGC and CTR in FR2 in late learning (*n*_CTR_ = 10, *n*_iuGC_ = 10). **G** Area under the curve for early learning and late learning. **H** Percentage of HR preference when animals fulfill the criteria of the test (75% of HR preference in FR2). **I** Success curve representing the time that each group required to fulfill the criteria of the test. **J** Number of incomplete trials during early and late learning. Error bars denote SEM. *or ^#^*p* ≤ 0.05; **p ≤ 0.01.

Previous data from our group has shown that iuGC animals display motivational deficits, together with alterations in dopaminergic transmission from the VTA to the NAc [22, 26]. To confirm this behavioral phenotype, iuGC and CTR animals were submitted to a PR schedule of reinforcement test (Fig. 1A) to evaluate their willingness to work for a food reward (pellet). During CRF training, both groups increased lever pressing throughout the days in a similar manner (F(1,19) = 0.2901, *p* = 0.5964, two-way ANOVA; Supplementary Fig. 1A). Likewise, all animals increased lever pressing in the FR schedule training (F(1,19) = 2.318, *p* = 0.1444 two-way ANOVA; Supplementary Fig. 1B), showing no learning impairments.

In the PR test day, iuGC rats displayed a significantly lower number of total lever presses (t(19) = 3.581, *p* = 0.0020; Fig. 1B), which was reflected in a lower number of food pellets earned (t(19) = 3.003, *p* = 0.0073; Fig. 1C). Concordantly, these results were reflected in a significantly lower breakpoint in comparison to the control group (57% decrease; t(19) = 3.118, *p* = 0.0057; Fig. 1D). These results confirmed that iuGC exposure causes significant motivational deficits towards pursuit of natural rewards.

### iuGC exposure negatively impacts effort-based decision-making

Next, we evaluated iuGC animals in an effort-based decision-making task [29]. We used an effort-discounting task (Fig. 1E), in which animals have available two levers, and pressing in one will result in the delivery of a high reward (HR; 4 food pellets), while pressing on the other will result in the delivery of a low reward (LR; 1 food pellet). To receive the HR, animals have to lever press increasingly more times under a FR schedule (2, 5, 10, or 20 times). To reach the criteria for end of training, one of the groups would have to display a preference for the HR on the FR2 schedule of 75% or above for three consecutive days.

In order to assess the evolution of behavioral performance throughout sessions (extended data Supplementary Fig. 1C–H), we combined data into early (days 1 and 2) and late (days 5 and 6) phases of training. Regarding the early phase of the task, no significant differences in performance between groups were observed (F(1,18) = 1.846, *p* = 0.1911 ANOVA; Fig. 1F); both iuGC and CTR rats presented similar levels of choice for the HR lever (FR2: *p* = 0.4363; FR5: *p* = 0.3771; FR10, FR20: *p* > 0.9999). However, in the late sessions, iuCG rats press significantly less the HR lever in comparison to control animals (F(1,18) = 5.470, *p* = 0.0311 ANOVA, Fig. 1F). The area under the curve of early learning was similar between groups (t(18) = 1.346, *p* = 0.1950; Fig. 1G), while on the last days of the test, iuGC animals presented reduced preference for the HR option (t(18) = 2.269, *p* = 0.0358; Fig. 1G).

After 6 days of test, the control group fulfilled the criteria for the end of the test (average of HR preference above 75% in the FR2 schedule during 3 consecutive days) (F(1,18) = 4.844, *p* = 0.0410 ANOVA; Fig. 1H). CTR animals showed a higher preference for HR in the FR2 schedule in comparison to iuGC rats (post hoc ANOVA *p* = 0.0376; Fig. 1H), suggesting that iuGC animals present reduced motivation when the effort cost increases.

We decided to continue the task with the iuGC group in order to observe if these animals would reach the test criteria if given more time. iuGC group presented a delay in their effort curve, since these animals required 7 additional days to achieve criterion performance (Log Rank (Mantel–Cox) X(21,22) = 4817, *p* = 0,028, Fig. 1I). In concordance, the duration to complete the required number of presses on the HR lever in early learning and late learning was increased in iuGC group in FR2 ratio (Supplementary Fig. 1I), indicating that when these animals selected the HR lever, they showed less response vigor compared to CTR rats.

We also analyzed the number of incomplete trials (the required number of lever presses was not reached within the 25 s trial period) in early and late sessions. IuGC group showed a higher number of incomplete trials in the late sessions in comparison with CTR rats (FR2: t(18) = 2.422, *p* = 0.0262; FR5: t(18) = 2.378, *p* = 0.0287; FR10: t(18) = 2.111, *p* = 0.0490; FR20: t(18) = 2.121, *p* = 0.0481; Fig. 1J); but not in the early sessions (FR2: t(18) = 2.032, *p* = 0.0572; FR5: t(18) = 1.574, *p* = 0.1238; FR10: t(18) = 0.3046, *p* = 0.7642; FR20: t(18) = 0.1455, *p* = 0.8859; Fig. 1J).

Overall, these data demonstrate that iuGC rats present a slower performance in the test, decreased preference for the high-effort/high-reward option, and higher number of incomplete trials, which is indicative of reduced motivation when the effort cost is high.

### Impaired neuronal activation of iuGC rats during effort-based decision-making

**Fig. 2:**
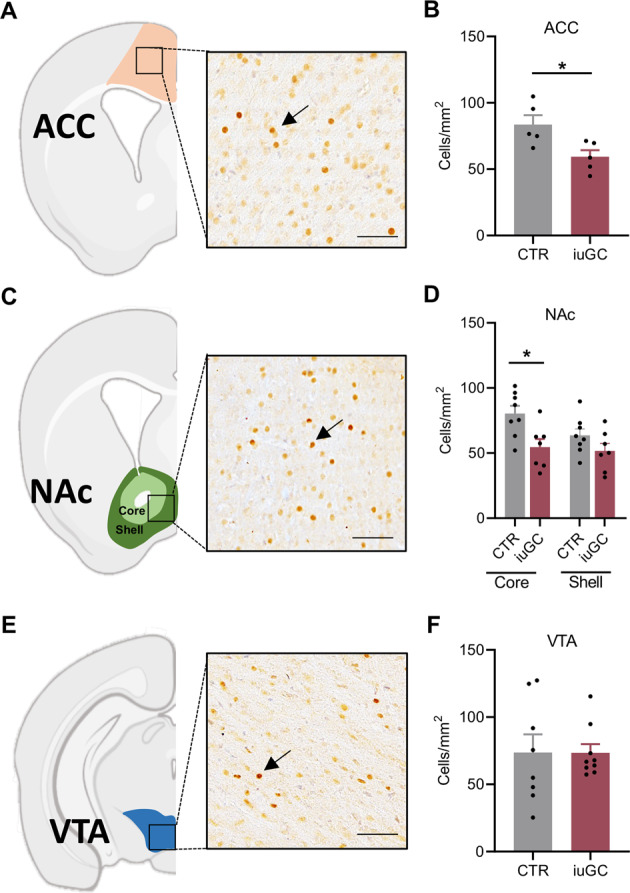
Neuronal recruitment impairment in the ACC and NAc in an effort-discounting task caused by iuGC exposure. **A** Representative immunohistochemistry of c-fos in the ACC (*n*_CTR_ = 5, *n*_iuGC_ = 6). **B** Effort-discounting task recruited ACC of both groups but at different extents, with the activation being significantly lower in iuGC rats. **C** Representative immunohistochemistry of c-fos in the NAc core (*n*CTR = 8, *n*_iuGC_ = 7) and shell (*n*_CTR_ = 8, *n*_iuGC_ = 7). **D** The NAc core of iuGC rats presented significantly less c-fos^+^ neurons than CTR rats upon task performance, but not **E** the NAc shell. **F** Representative immunohistochemistry of c-fos in the VTA (*n*_CTR_ = 8, *n*_iuGC_ = 8). **G** No differences in the number of c-fos^+^ neurons were observed in the VTA. Error bars denote SEM. *p ≤ 0.05. Scale bar = 25 μm.

To better determine the impact of iuGC exposure on neural circuits related to motivation, and the relevance of such changes on behavioral performance, we assessed neuronal recruitment during effort-based decision-making task by performing immunohistochemistry against the protein encoded by the immediate early gene c-fos (Fig. 2A). We counted c-fos^+^ cells in the ACC, NAc, and VTA due to the involvement of these brain regions in effort-based motivated behaviors [13, 42, 43].

Briefly, animals were subjected to the effort-based decision-making task and were sacrificed 60 min after the end of the test. IuGC animals presented a significantly lower number of c-fos^+^ cells in the ACC in comparison to CTR rats (Fig. 2B; t(8) = 2.760, *p* = 0.0247). Analysis of the rostrocaudal distribution of c-fos^+^ cells in both groups showed no significant differences (Supplementary Fig. 2A). iuGC animals also presented a significantly lower number of c-fos^+^ cells in the NAc core (Fig. 2C; t(13) = 2.950, *p* = 0.0113), which was reflected across the entire rostrocaudal distribution of c-fos^+^ cells (F (1,13) = 5.053, *p* = 0.0426; Post Hoc: *p*(2.52–2.16) > 0.999, *p*(2.08–1.80) = 0.0858, *p*(1.68–1.32) = 0.0896, *p*(1.20–0.96) > 0.9999; Supplementary Fig. 2B). Yet, no differences were found in the NAc shell (Fig. 2D; t(13) = 1.549, *p* = 0.1453; Supplementary Fig. 2C). Finally, no alterations in the recruitment of neurons were found in the VTA (Fig. 2E; t(15) = 0.01780, *p* = 0.9860; Supplementary Fig. 2D).

Interestingly, a positive correlation between the number of c-fos^+^ cells and effort behavior (*R*^2^ = 0.3599, *p* = 0.0181; Supplementary Fig. 3B) was observed in the NAc core, no significant changes were observed in the NAc shell, ACC, or VTA (Supplementary Fig. 3A, C, D).

These results show that iuGC rats present a significant decrease in the number of c-fos^+^ cells in the ACC and the NAc in comparison with CTR rats after the performance of an effort-based decision-making task.

### IuGC exposure leads to functional changes in the ACC-NAc circuitry

Based on the c-fos analyses, that showed reduced neuronal recruitment in the ACC and NAc, we next performed electrophysiological recordings in the ACC, NAc, and VTA, in order to understand if the alteration in neuronal recruitment could be related with impaired neuronal activity. We evaluated both basal and evoked activity of these brain regions using in vivo electrophysiological extracellular recordings in anesthetized animals.

**Fig. 3:**
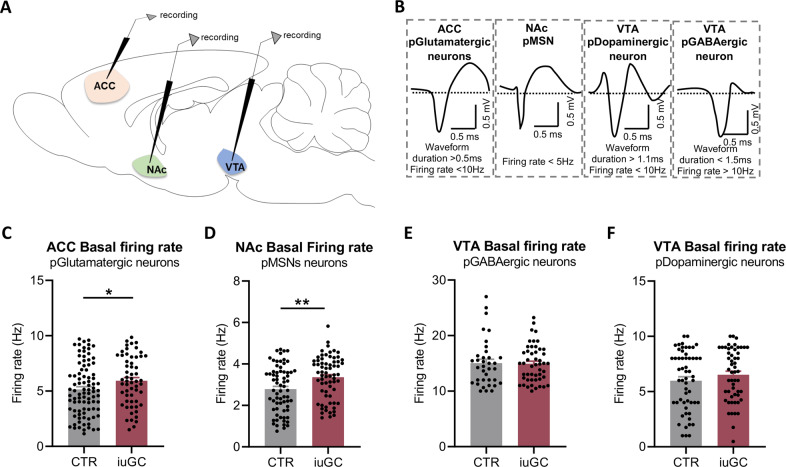
iuGC exposure hampers neuronal basal activity of ACC and NAc. **A** Schematic representation of electrophysiological recordings of ACC, NAc, and VTA. **B** ACC neurons were separated according to firing rate and waveform characteristics: putative glutamatergic neurons; NAc neurons were separated according to firing rate and waveform characteristics putative medium spiny neurons (pMSNs); VTA neurons were classified as: putative dopaminergic neurons or putative GABAergic neurons. **C** The basal firing rate of the ACC is significantly different between groups, with iuGC rats presenting a hyperactivation of putative glutamatergic neurons. *n*_CTR_ = 4 animals/90 cells (12–32 cells/animal); *n*_iuGC_ = 4 animals/58 cells (13–18 cells/animal). **D** iuGC group has an hyperactivation of putative MSNs of the NAc; *n*_CTR_ = 4 animals/68 cells (8–16 cells/animal); *n*_iuGC_ = 5 animals/67 cells (6–20 cells/animal). The basal activity of the VTA of **E** putative GABAergic (*n*_CTR_ = 4 animals/56 cells (2–12 cells/animal); *n*_iuGC_ = 4 animals/54 cells (2–15 cells/animal)) and **F** putative dopaminergic neurons (*n*_CTR_ = 5 animals/36 cells (2–12 cells/animal); *n*_iuGC_ = 5 animals/55 cells (2–12 cells/animal)) is similar between groups. Error bars denote SEM. *p ≤ 0.05; **p ≤ 0.01.

In comparison to CTR group, iuGC treated rats presented increased basal activity of ACC putative glutamatergic neurons (16% increase; t(145) = 2.005, *p* = 0.0468; Fig. 3C). No differences were found in ACC interneurons’ basal activity between groups (Supplementary Fig. 4A). Similarly, basal activity of NAc putative MSNs (pMSNs) was increased in iuGC animals in comparison to CTR rats (Increase of 24%; t(133) = 3.086, *p* = 0.0025; Fig. 3D). However, no differences were observed in NAc interneurons’ activity (Supplementary Fig. 4B).

Conversely, no differences between groups were found in the basal activity of VTA putative dopaminergic (pDopaminergic) neurons (t(108)=1.107, *p* = 0.2707; Fig. 3F) or VTA putative GABAergic (pGABAergic) neurons (t(83) = 0.13196, *p* = 0.8953; Fig. 3E).

**Fig. 4:**
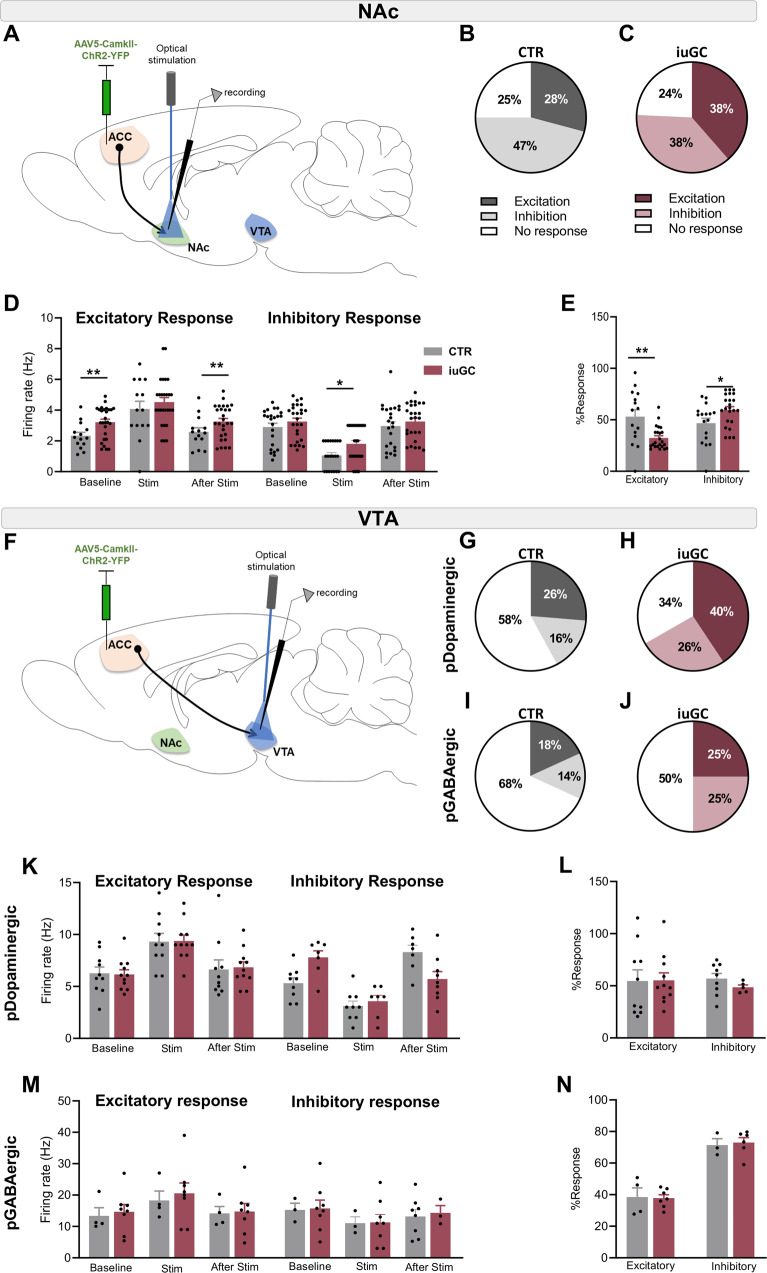
Distinct NAc and VTA neuronal response to ACC optogenetic stimulation in iuGC animals. **A** Strategy used for optogenetic activation of ACC projecting neurons in the NAc and VTA. Response of NAc neurons to ACC optogenetic stimulation in **B** CTR rats **C** and iuGC rats (*n*_CTR_ = 5 animals/48 cells (10–20 cells/animal); *n*_iuGC_ = 5 animals/72 cells (12–22 cells/animal). **D** Change in neuronal activity of NAC neurons upon ACC optical activation. **E** The NAc of iuGC rats present impaired magnitude of response, to ACC stimulation. **F** Strategy used for optogenetic activation of ACC projecting neurons in the VTA. Response of VTA pDopaminergic (*n*_CTR_ = 4 animals/20 cells (2–10 cells/animal); *n*_iuGC_ = 4 animals/36 cells (3–10 cells/animal) and pGABAergic neurons (*n*_CTR_ = 4 animals/23 cells (4–12 cells/animal); *n*_iuGC_ = 4 animals/32 cells (1–7 cells/animal) to ACC optogenetic stimulation in (**G**, **I)** CTR rats (**H**, **J**) and iuGC rats, respectively. Optical stimulation of ACC terminals in the VTA does not alter firing rate of **K** pDopaminergic neurons or **M** pGABAergic neurons. The magnitude of response of **L** pDopaminergic neurons or **N** pGABAergic neurons is similar between groups. Error bars denote SEM. *p ≤ 0.05; **p ≤ 0.01.

As the ACC projects to both NAc and VTA, we next decided to evaluate the evoked activity of NAc and VTA in response to ACC optical stimulation. To achieve this, we injected a construct containing channelrhodopsin (ChR2) in fusion with the enhanced yellow fluorescent protein (eYFP) under the control of the CAMKII minimal promoter (AAV5-CAMKII-ChR2-YFP; Fig. 4A) in the ACC of iuGC and CTR rats. To record optogenetically-evoked activity upon ACC terminals activation (473 nm; 20 Hz, 5 ms pulses over 1 s), an optical fiber coupled with a tungsten electrode was placed in the NAc or in the VTA (Fig. 4A, F).

In CTR animals, 28% of NAc cells increased firing rate in response to ACC terminal stimulation, 47% were inhibited and 25% did not change their activity in comparison to baseline (Fig. 4B). In the iuGC group (Fig. 4C), 38% of the NAc cells were excited upon ACC terminal stimulation, 38% presented a decrease in their activity and 24% did not change.

For cells that presented an excitatory response, iuGC group presented a significantly higher basal firing rate in comparison to CTR rats (t(39) = 2.719, *p* = 0.0097, Fig. 4D). During stimulation, the response was similar between groups (t(39) = 0.8095, *p* = 0.4231; Fig. 4D, Supplementary Fig. 5A). Cells that presented an inhibitory response during the stimulation period presented a higher firing rate in iuGC animals in comparison to CTR group (t(45) = 2.483, *p* = 0.0168; Fig. 4D).

Because NAc neurons recorded from iuGC animals presented a basal firing rate higher than CTR animals (Fig. 4D), we evaluated the response magnitude of each type of response (percentage of increase or decrease in relation to baseline; Fig. 4E). iuGC animals presented a lower response magnitude in excited cells (t(36) = 2.726, *p* = 0.0098; Fig. 4E), and a higher response magnitude in inhibited cells (t(38) = 2.344, *p* = 0.0244; Fig. 4E). The firing rate of cells that presented no change in activity was higher in iuGC animals (t(28) = 4097, *p* = 0.0003, Supplementary Fig. 5D). These results suggest that there is an imbalance in the excitatory and inhibitory inputs from the ACC to the NAc of iuGC rats.

Regarding ACC-evoked VTA activity, in CTR group, 26% of pDopaminergic neurons presented increased firing rate upon ACC terminal stimulation (Fig. 4G), 16% decreased (Fig. 4G) and 58% did not respond to stimulation. In the iuGC group, 40% of pDopaminergic neurons increased activity (Fig. 4H), 26% decreased and 34% did not alter the firing rate. Subdivision of cells into excited, inhibited and no change showed no differences in VTA pDopaminergic activity of iuGC and CTR rats upon stimulation of ACC terminals (Fig. 4L, M and Supplementary Fig. 5B, E).

Regarding VTA pGABAergic cells, in the CTR group, 18% of the neurons increased activity, 14% decreased and 68% did not respond to optical stimulation of ACC-VTA terminals (Fig. 4I). Similar to controls, iuGC rats presented a decrease in activity of 25% of pGABAergic neurons, while 25% increased and 50% did not respond (Fig. 4J). pGABAergic neurons of iuGC rats seem to respond to stimulus similarly to CTR rats (Fig. 4M, N and Supplementary Fig. 5C, F).

Overall, these data suggest that iuGC exposure changes ACC and NAc neuronal activity, and significantly impairs ACC-NAc connectivity, that could account for, at least in part, the impairments observed in the motivation and the effort discounting behavioral tasks.

## Discussion

Prenatal stress or increased GC exposure has been shown to induce long-lasting neurobiological alterations in the reward circuit [22, 27, 44], leading to the emergence of motivational deficits later in life [2, 45, 46]. Herein, we show that animals exposed *in utero* to increased levels of GCs present motivational deficits in adulthood, as evaluated in the PR task. We also show that iuGC animals display reduced preference for larger rewards associated with a greater effort cost compared to CTR animals. These findings are in line with the observed hypodopaminergic state previously reported in iuGC rats [23, 47–49]. Indeed, several studies have shown that rats with impaired DA transmission reallocate their instrumental behavior away from food-reinforced tasks with high response requirements [16, 50–52].

IuGC exposure was shown to alter neuronal recruitment during the effort discounting task, since both ACC and NAc core, but not shell had reduced number of c-fos^+^ cells. This data is in concordance with other studies, where inactivation of the NAc core, but not the shell, via infusion of GABA A/B agonists muscimol/baclofen reduced preference for a high-effort option [43, 53]. In fact, the NAc core has long been implicated in several aspects of instrumental action, a mechanism that is dependent on glutamatergic and dopaminergic transmission [53–57]. Regarding the ACC, it is known that this brain region plays an integral role in biasing certain forms of cost/benefit decision-making [58], particularly for the evaluation of the expected benefits and costs associated with each potential choice of action during a decision [19]. In line, previous studies report that ACC integrity is necessary for proper effort-based decision-making [42, 59, 60]. Likewise, inactivation of both ACC and NAc lead to reallocation of animals’ choice towards low reward options [53]. Thus, the reduced preference for larger rewards associated with a greater effort cost induced by iuGC is likely due, at least in part, to perturbations in neural activity within ACC-NAc circuit.

In contrast to the effects of iuGC on ACC-NAc activity, when analyzing the VTA recruitment, no group differences were observed in the number of c-fos^+^ cells after the effort task, though a hypodopaminergic state has been reported in iuGC rats [22, 23]. In agreement, basal activity of VTA is not altered in iuGC rats, as evaluated by our electrophysiological recordings, and as described in previous work using this animal model [46]. Thus, although VTA-mediated DA release plays a role in cost encoding [61], influencing effort decisions, it is also known that DA release can occur independently of cell body spiking activity [14]. In fact, NAc core dopamine fluctuations associated with motivation can occur independently from VTA dopamine cell firing [14]. Thus, the lack of effect of iuGC on this marker of VTA DA neural activity does not necessarily preclude the possibility that this treatment may have perturbed DA release at terminal regions.

Given that performance of the effort task was associated with decreased c-fos labeling in the ACC and NAc core in iuGC rats, we decided to evaluate neuronal activity of the ACC and evoked activity of the NAc (and VTA). Somewhat counterintuitively, we found that basal firing rate of the ACC is significantly increased in comparison to the control group. Others have shown that ACC activity encodes a relative, integrated cost-benefit representation of available choice options that is optimized to create the ideal, most balanced effort/outcome, result [62]. In addition, chemogenetic activation of ACC results in disrupted lever pressing for a higher value choice [60], as does pharmacological disinhibition of the dorsomedial prefrontal cortex [63]. Thus, these data suggest a mechanism by which the ACC tightly regulates effortful decisions. In accordance, human studies performed in depressed patients showed that in these individuals, choosing between two reward options was associated with ACC hyperactivation during reward selection [64, 65]. Collectively, these data suggest that excessive ACC activity, induced either by artificial activation, by disease, or by exposure to increased iuGC levels as we report here, can perturb effort-related cost/benefit decisions and bias choice away from rewards that are more preferred but also more difficult to obtain.

In addition, we also assessed the activity of the NAc and observed a similar increase in basal firing activity in iuGC animals. Importantly, within the NAc, a unique sub-set of neurons exhibit activity increases that are preferential for low-cost options over high-cost options, showing that there is a population bias towards low-cost choices in effort-based decision making [66]. In addition, a human study using functional magnetic resonance, showed that activity in the ventral striatum signaled the expected amount of reward discounted by the amount of effort to be invested [67]. Overall, these results indicate that the NAc neurons are encoding some aspects of effort decision-making. Thus, the observed changes in NAc activity in iuGC animals can also contribute to impaired effort-dependent responses.

Individual NAc neurons receive diverse cortical inputs, including from the ACC [68]. The NAc is known to process reward-related information [69, 70], and its outputs [21, 43, 68] are critical regulators of decisions that involve effort. Previous studies have also indicated that ACC-NAc circuit plays a role in mediating reward approach behavior [21]. Thus, given that both ACC and NAc presented functional impairments in iuGC rats, we next decided to test if ACC-evoked NAc responses were altered in iuGC animals. For that, we optogenetically activated ACC terminals in the NAc and showed an imbalance in the evoked responses in NAc pMSNs. We observed decreased magnitude of NAc excitatory responses and increased magnitude of inhibitory responses. It is known that in patients suffering from schizophrenia, these individuals present NAc hypofunction that is manifested during effort-based decisions, reflecting dimensional motivation impairment [71, 72].

In this work, we showed that iuGC exposure causes an impairment in effort-based decision-making. We observed reduced c-fos staining in the ACC and NAc core after behavioral performance. Electrophysiological data showed a basal increase in the activity of ACC and NAc neurons (but not VTA), and impaired ACC-evoked NAc response. Further studies are needed to understand how iuGC exposure triggers these long-lasting alterations in the ACC-NAc circuitry.

It is important to mention that this model *mimic*s the administration of synthetic GCs between weeks 26–34 of gestation to women in risk of preterm labor. In the rat iuGC model, pregnant dams are exposed to DEX on days 18 and 19 of gestation which corresponds to a similar period of gestation in humans. Though this GC administration is lifesaving, there is also evidence showing a deleterious effect in the developing brain. For example, in humans, prenatal GC exposure is associated with decreased thickness of the ACC in 6–10 year old children [73]. In another human study, in a task designed to evaluate cognitive conflict monitoring, there was a reduction in the activation of the fronto-parietal network, which underlies cognitive and behavioral control, most notably in the cingulate of iuGC-exposed adolescents [74]. In accordance to human studies, previous work from our team showed that iuGC animals present decreased ACC volumes in comparison to control animals [22]. Here, we now report that iuGC animals also present ACC functional differences associated with impaired effort.

Overall, these data suggests that the ACC is affected both in humans and rodent models of iuGC exposure, strengthening our findings.

Understanding how stress and glucocorticoid exposure during early development can affect ACC-NAc circuitry that regulates motivation and cost/benefit decision-making can provide new insights into the neural basis of effort responses, and better comprehend disorders characterized by aberrant effort-related processes such as depression or addiction.

## Supplementary information

Supplementary Data

## Data Availability

The data presented in this study are available upon request from the corresponding authors.
