# Supplementary material for: Prenatal dexamethasone exposure alters effort decision making and triggers nucleus accumbens and anterior cingulate cortex functional changes in male rats
Source: Transl Psychiatry. 2022 Aug 19;12:338. doi: 10.1038/s41398-022-02043-4 (PMC9391327; doi:10.1038/s41398-022-02043-4)
Supplement: Supplementary file 1 — Supplementary Data [file 41398_2022_2043_MOESM1_ESM.pdf]

Supplementary Information

Prenatal glucocorticoid exposure alters effort decision making and triggers nucleus accumbens and anterior cingulate cortex functional changes

DOI: https://doi.org/10.1038/s41398-022-02043-4

Ana Verónica Domngues[1,2], MSc, Barbara Coimbra[1,2], PhD, Raquel Correia[1,2], MSc, Catarina Deseyve[1,2], BSc, Stan B Floresco[3], PhD, Nuno Sousa[1,2,4], MD PhD, Carina Soares Cunha[1,2]*, PhD, Ana João Rodrigues[1,2,4]* , PhD

1-Life and Health Sciences Research Institute (ICVS), School of Medicine, University of Minho, Braga, Portugal.
2- ICVS/3B's-PT Government Associate Laboratory, Braga/Guimarães, Portugal.
3- Department of Psychology and Djavad Mowafaghian Centre for Brain Health, University of British Columbia, 2136 West Mall, Vancouver, BC V6T 1Z4, Canada.
5- Clinical Academic Center-Braga (2CA), Braga, Portugal

***Correspondence:**
Ana João Rodrigues
Email: ajrodrigues@med.uminho.pt
Tel:+351253604929

&

Carina Soares-Cunha
Email: carinacunha@med.uminho.pt
Tel:+351253604831

Supplementary Figures

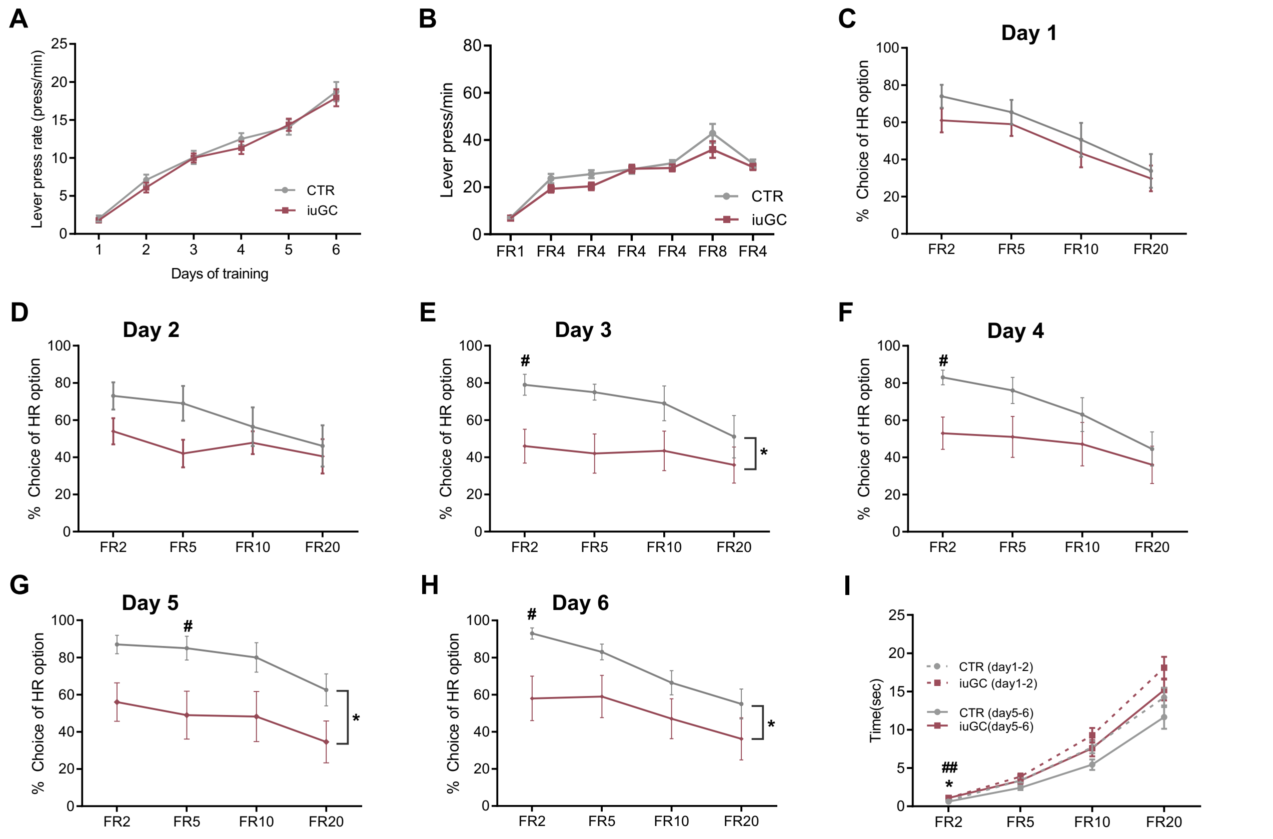

Supplementary Figure 1. iuGC exposure impairs motivated behavior. A Continuous Reinforcement (CRF) training sessions of the Progressive Ratio (PR) test. **B** Fixed Ratio (FR) training sessions of the PR test. **C** First day of effort task (F(1,18)=0.8485, p=0.3692; Post-Hoc: p(FR2)=0.6456, p(FR5)>0.9999, p(FR10)>0.9999, p(FR20)>0.99999). **D** Day 2 of effort task (F(1,18)=2.019, p=0.1725; Post-Hoc: p(FR2)=0.3090, p(FR5)>0.1487 p(FR10)>0.9999, p(FR20)>0.99999). **E** Day 3 of effort task (F(1,18) =5.011, p=0.0381; (F(1,18)=2.019, p=0.1725; Post-Hoc: p(FR2)=0.0303, p(FR5)>0.0532 p(FR10)>0.3529 p(FR20)>0.99999). **F** Day 4 of effort task (F(1,18)=2.953, p=0.1028; (F(1,18)=2.019, p=0.1725; Post-Hoc: p(FR2)=0.0325, p(FR5)>0.2973 p(FR10)>0.9999, p(FR20)>0.99999). **G** Day 5 of effort task (F(1,18)=6.201, p=0.0228; (F(1,18)=2.019, p=0.1725; Post-Hoc: p(FR2)=0.1188, p(FR5)>0.0482 p(FR10)>0.1039, p(FR20)>0.1974). **H** Day 6 of effort task (F(1,18)=4.833, p=0.412; (F(1,18)=2.019, p=0.1725; Post-Hoc: p(FR2)=0.0306, p(FR5)>0.2333 p(FR10)>0.4330, p(FR20)>0.4677). **I** Average trial duration during early learning and late learning of the effort discounting task. Error bars denote SEM. *or $^{\#}p \leq 0.05$.

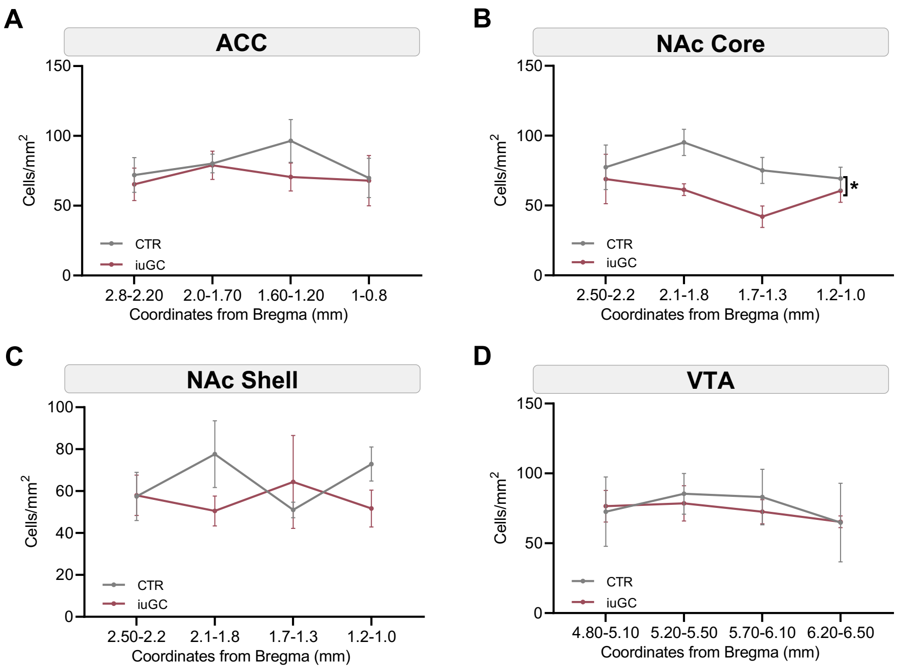

Supplementary Figure 2. C-fos+ cell countings along the rostracaudal axis of A ACC (n_{CTR}=5, n_{iuGC}=5; F(1,8)=0.3958, p=0.5468). **B** NAc core (n_{CTR}=8, n_{iuGC}=7; (F(1,13)=5.053, p=0.0426; Post-Hoc: p(2.50-2.2)>0.999, p(2.1-1.8)=0.0858, p(1.7-1.3)=0.0896, p(1.2-1.0)>0.9999)) and **C** NAc shell (F(1,13)=0.4871, p=0.4975; Post-Hoc: p(2.50-2.2)>0.9999, p(2.1-1.8)>0.9999, p(1.7-1.3)>0.9999, p(1.2-1.0)=0.6201). **D** VTA (n_{CTR}=8, n_{iuGC}=9; F(1,15)=0.03107, p=0.8624). Error bars denote SEM. *$p \leq 0.05$.

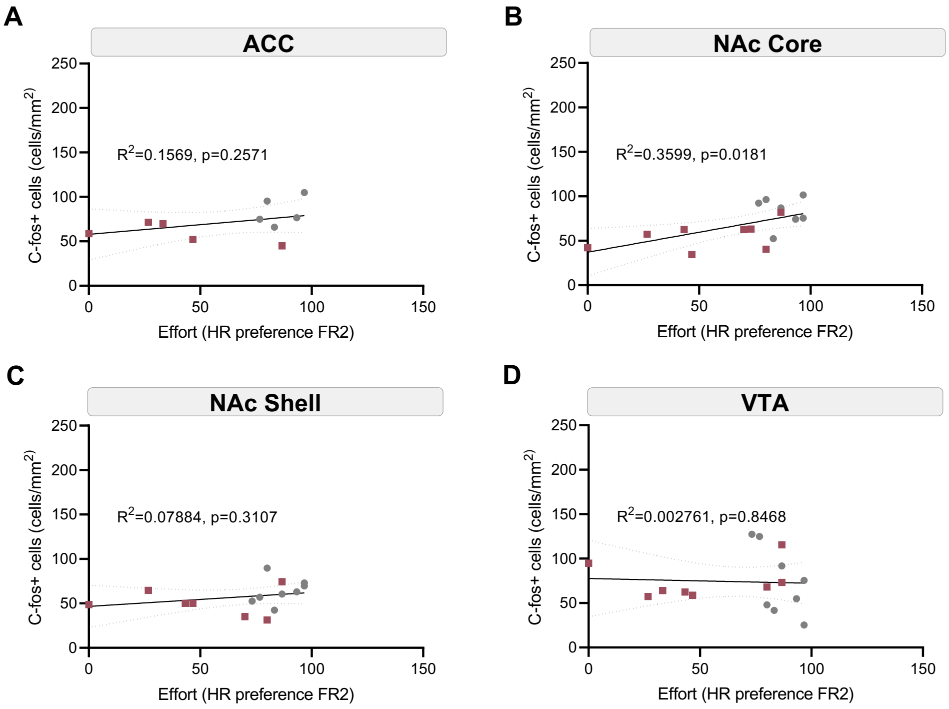

Supplementary Figure 3. Correlation of behavioral performance in effort task with c-fos countings in the A ACC (nCTR=5, niuGC=5), **B** NAc Core (nCTR=7, niuGC=8) and **C** NAc Shell (nCTR=7, niuGC=8), **D** VTA (nCTR=8, niuGC=8). Simple linear regression.

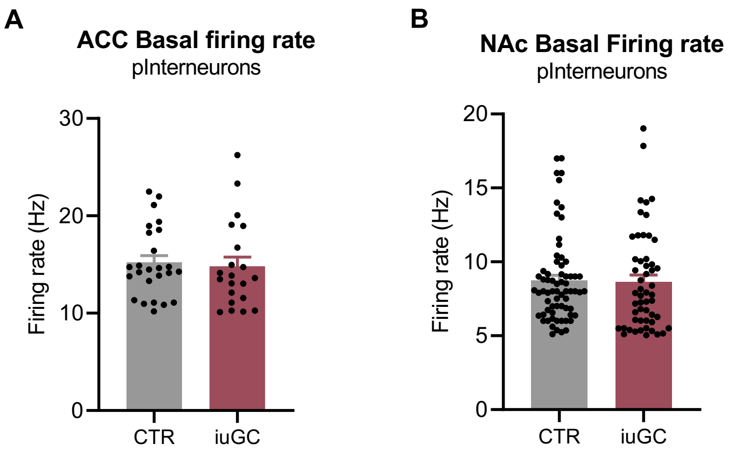

Supplementary Figure 4. iuGC exposure does not impact the activity of interneurons of the ACC and NAc. A Basal firing rate of ACC putative interneurons (pInterneurons). **B** Basal firing rate of NAc pInterneurons. Error bars denote SEM.

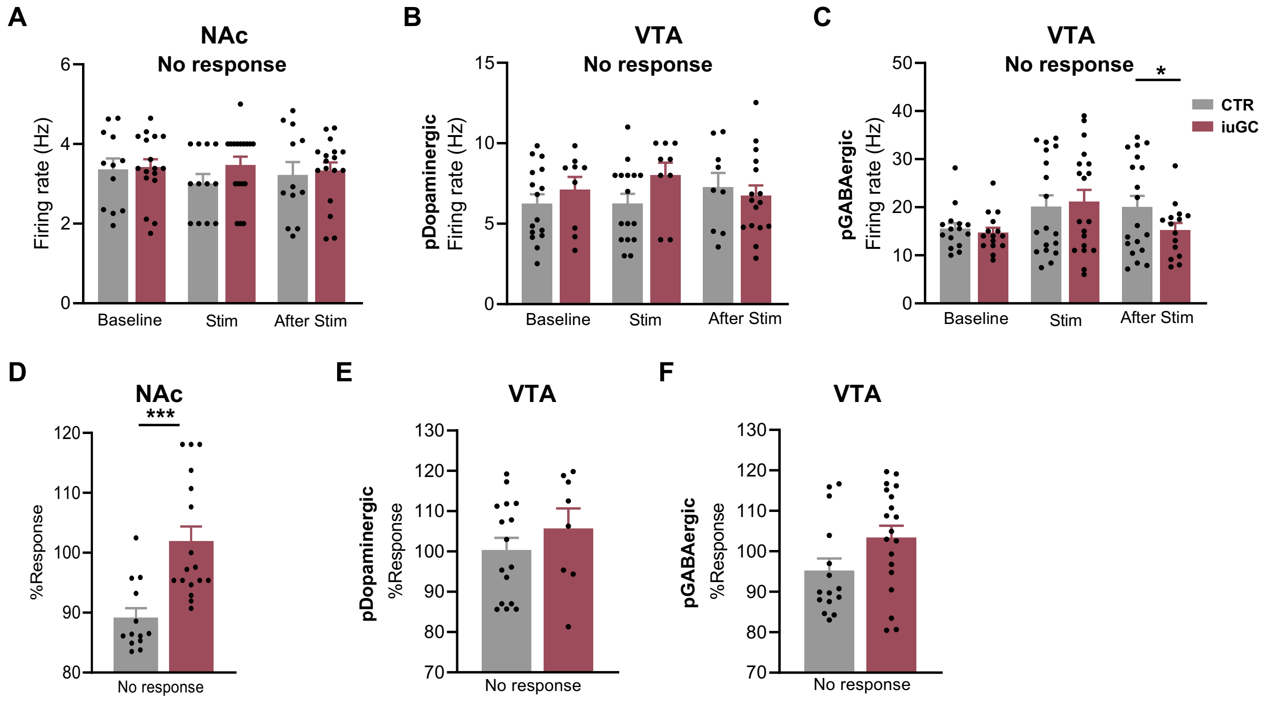

Supplementary Figure 5. Spontaneous activity of cells that do not respond to ACC terminal stimulation. A Firing rate of NAc non-responsive cells during baseline, stimulation and after stimulation. **B** Firing rate of VTA putative dopaminergic (pdopaminergic) non-responsive cells during baseline, stimulation and after stimulation. **C** Firing rate of VTA putative GABAergic (pGABAergic) non-responsive cells during baseline, stimulation and after stimulation. The NAc of iuGC rats **D** presents impaired magnitude of response to ACC stimulation. The magnitude of response of **E** pDopaminergic neurons or **F** pGABAergic neurons is similar between groups. Error bars denote SEM.
